# Human papillomavirus E5 protein, the undercover culprit of tumorigenesis

**DOI:** 10.1186/s13027-018-0208-3

**Published:** 2018-11-09

**Authors:** Nima Hemmat, Hossein Bannazadeh Baghi

**Affiliations:** 1 Immunology Research Center, Tabriz University of Medical Sciences, Tabriz, Iran; 2 Drug Applied Research Centre, Tabriz University of Medical Sciences, Tabriz, Iran; 3 Infectious and Tropical Diseases Research Center, Tabriz University of Medical Sciences, PO Box 5165665931, Tabriz, Iran; 4 Department of Microbiology Faculty of Medicine, Tabriz University of Medical Sciences, Tabriz, Iran

**Keywords:** HPV, Cervical cancer, HPV E5 protein, Tumorigenesis

## Abstract

Human papillomavirus (HPV) is the most common viral infection of the reproductive tract worldwide. It has been well documented that the HPV oncoproteins E6 and E7 play important roles in cancer progression and maintenance. However, the high risk HPV E5 protein is also demonstrated to affect some cellular pathway and signaling in human cell lines. In this letter we argue for the need of further investigation and suggest that the HPV E5 protein should be acknowledged as an oncoprotein of HPV.

## Letter to the editor

**Fig. 1:**
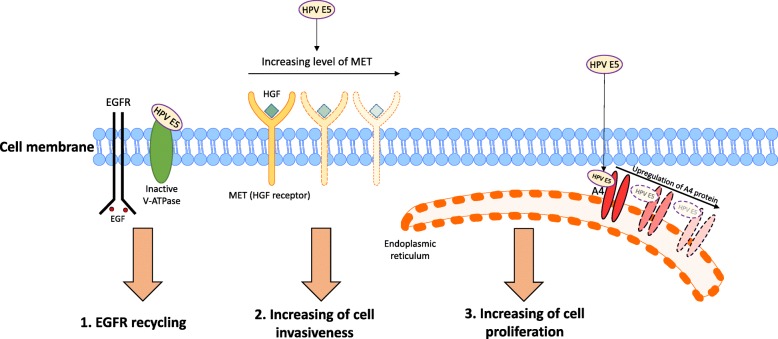
Effect of HPV E5 protein on cellular pathways

Dear Editor,

Human papillomavirus (HPV) has been proven to be the main cause of cervical cancer worldwide [1]. Most studies, about HPV tumorgenesis, focus on the role that high risk HPV E6, and E7 proteins play [2]. However high risk HPV E5 protein, one of the virus early phase proteins, is demonstrated to have an important effect on cellular and signaling pathways in human cell lines [3]. Many functions have been described for this viral protein, including, cell transforming activity (Fig. 1), influencing cell cycle and growth factors, induction of apoptosis and endoplasmic reticulum (ER) stress, and immune evasion [3]. HPV E5, as a cell transformer, can interact with the 16 KDal subunit of vacuolar-ATPase (V-ATPase) and disrupt acidification of endosomes [4]. This phenomenon enhances epidermal growth factor (EGF) receptor recycling [4]. Additionally, it has been indicated that the E5 protein increases the expression level of Met, a hepatocyte growth factor (HGF) receptor, promoting transformed cell invasiveness [5]. E5 is also shown to be bonded with an A4 protein, a transmembrane lipoprotein of the endoplasmic reticulum, thus regulating proliferation of infected cells [6]. With all of this considered, it is highly suggested that the HPV E5 protein should be acknowledged as an oncoprotein of HPV. Especially, for the production of DNA-based vaccines this can be of utmost importance. As HPV infections are spread more widely around the world [7], also affecting areas that were thought to be protected by it due to more conservative sexual conduct [8], DNA-based vaccines against HPV should contain not only HPV E6 and E7 coding genes [9], but also E5. Novel vaccines could be used therapeutically as well as in a preventive way. Currently, dispensed vaccines are based on the HPV L1 capsid protein and are able to induce protective immunity (by production of memory cells against L1).

## Abbreviations

EGF: Epidermal growth factor
ER: Endoplasmic reticulum
HGF: Hepatocyte growth factor
HPV: Human papillomavirus
V-ATPase: Vacuolar-ATPase

## References

[1] Wakeham K, Kavanagh K (2014). The burden of HPV-associated anogenital cancers. Curr Oncol Rep.

[2] Ganguly N, Parihar SP (2009). Human papillomavirus E6 and E7 oncoproteins as risk factors for tumorigenesis. J Biosci.

[3] Venuti A, Paolini F, Nasir L, Corteggio A, Roperto S, Campo MS (2011). Papillomavirus E5: the smallest oncoprotein with many functions. Mol Cancer.

[4] Kim M-K, Kim HS, Kim S-H, Oh J-M, Han JY, Lim JM (2010). Human papillomavirus type 16 E5 oncoprotein as a new target for cervical cancer treatment. Biochem Pharmacol.

[5] Scott ML, Coleman DT, Kelly KC, Carroll JL, Woodby B, Songock WK (2018). Human papillomavirus type 16 E5-mediated upregulation of met in human keratinocytes. Virology.

[6] Halavaty KK, Regan J, Mehta K, Laimins L (2014). Human papillomavirus E5 oncoproteins bind the A4 endoplasmic reticulum protein to regulate proliferative ability upon differentiation. Virology.

[7] Baseman JG, Koutsky LA (2005). The epidemiology of human papillomavirus infections. J Clin Virol.

[8] Baghi HB, Yousefi B, Oskouee MA, Aghazadeh M (2017). HPV vaccinations: a middle eastern and north African dilemma. Lancet Infect Dis.

[9] Ahn J, Peng S, Hung CF, Roden R, Wu TC, Best SR (2017). Immunologic responses to a novel DNA vaccine targeting human papillomavirus-11 E6E7. Laryngoscope.

